# Differences in microbial diversity and environmental factors in ploughing-treated tobacco soil

**DOI:** 10.3389/fmicb.2022.924137

**Published:** 2022-09-12

**Authors:** Yuzhen Zhang, Guodong Bo, Minchong Shen, Guoming Shen, Jianming Yang, Shanyu Dong, Zhaohe Shu, Zhaobao Wang

**Affiliations:** ^1^Energy-Rich Compounds Production by Photosynthetic Carbon Fixation Research Center, Shandong Key Laboratory of Applied Mycology, College of Life Sciences, Qingdao Agricultural University, Qingdao, China; ^2^Tobacco Research Institute of Chinese Academy of Agricultural Sciences, Qingdao, China; ^3^Yichang Tobacco Company of Hubei Province, Yichang, China; ^4^Enshi Tobacco Company of Hubei Province, Enshi, China

**Keywords:** tobacco soil, environmental factors, enzymatic activity, microbial diversity, deep-plowing, spatial differences

## Abstract

During agricultural production, plowing affects the existing traits of the planted soil, including environmental factors (physicochemical properties and soil enzymatic activity) and microbial community, but whether deep tillage and conventional tillage cause differences in soil microecology are unknown. In this study, the 16S rRNA high-throughput sequencing technology was combined with soil environmental factor detection to analyze the differences in microbial diversity of smokey soils at different depths. As a result, the composition and structure of microbial community varied in different soil depth after plowing. Two dominant phyla, Actinobacteria and Acidobacteria, have varied a lot between the deep-plowing treatment HS3 (the sample in 10–20 cm depth after deep-plowing treatment) sample and the conventional tillage HC3 (treatment the sample in 10–20 cm depth after conventional tillage) sample. The abundance of Actinobacteria has increased significantly, while the abundance of Acidobacteria has decreased significantly. Moreover, deep tillage increased the activity of sucrase (S-SC) and nitrate reductase (NR) in samples with soil depth below 20 cm. In summary, deep tillage disturbed spatial microbial diversity and environmental factors significantly. This would provide new guidance for improving farmland management strategies, optimizing the activation methods of soil layers, further improving crop planting soil, and increasing crop yield.

## Introduction

During the whole process of plant growth, soil plays a vital role in providing substrate and nutrients. Soil with good fertility and appropriate microbial community structure is the basis for the healthy growth and development of plants (Van Bruggen et al., 2019; Khatoon et al., 2020). Soil environment, soil microorganisms, and crops could form a system of mutual restriction and promotion (Hartman and Tringe, 2019). Studies have shown that the microbial community structure in soil interacts with environmental factors (including physicochemical properties and enzymatic activities) and plays an important role in soil nutrient transformation and humus formation, meanwhile, the microbial diversity and variability reflect soil quality to a certain extent (Schloter et al., 2017; Afzaal et al., 2018). Moreover, the enzymes produced by microorganisms in soil can participate in soil structure improvement directly or indirectly. These demonstrated that soil microorganisms are one of the key factors driving plant–soil nutrient cycling (Barea et al., 2005; Lau and Lennon, 2012). Reversely, soil disturbance also has a certain influence on soil microbial diversity in crop cultivation, leading to the dynamic change in soil microbial community structure, and thereby affecting soil enzyme activities. At the same time, external factors or multiples of treatment means can also raise significant differences in the interaction between environmental factors and microbial community. For example, peanut continuous cropping tends to reduce soil microbial activity and destroy microbial community structure, which may be the main reason for continuous cropping failure (Li et al., 2014). Our previous studies have confirmed that environmental factors and the microbial community structure changed significantly along with the growth stage of tobacco (Wang et al., 2019). Moreover, improving soil carbon can increase the aroma of tobacco leaves, increase the abundance of *Lactobacillus* and *Marine spirulina*, and the revelation of the correlation between soil carbon, microbial community structure, and tobacco quality indicating the importance of improving soil carbon to improve tobacco quality (Shen and Glab, 2020). The bacteriostatic soil can inhibit the occurrence of soil-borne diseases by promoting the growth of beneficial bacteria and inhibiting the growth of potential pathogens (Ding et al., 2021).

In practical agricultural production, agricultural management practices affect soil conditions and the structure of soil microbial community and create a niche environment conducive to the growth of some soil microorganisms (Ashworth et al., 2017; Chamberlain et al., 2020). In soil ecosystems, tillage ensures a clean seedbed for early crop growth by reducing compaction, improving soil permeability, and eliminating competition (Halvorson et al., 2006; Behnke et al., 2018; Daigh et al., 2018). As reported, tillage, as one of the agricultural management methods, could increase crop yield by creating a more favorable growing environment for corn and soybean (Behnke et al., 2018, 2021). Therefore, appropriate tillage methods can optimize the agroecosystems to achieve higher crop yield and sustainable development of land utilization. In recent years, studies have been focused on revealing the changes in environmental factors and soil microbial diversity, and their relationship raised by soil disturbance treatments, during which tillage treatment was a traditional soil activation technology. It was demonstrated that short-term rice straw regression and tillage practice significantly changed the relative abundance and function of soil microbial community (Xia et al., 2019). In the study of wheat and maize rotation, it was found that the effect of tillage on the depth decay of bacterial community was significantly smaller than that under no tillage, and the depth decay of fungal community was significantly greater under reduced tillage than that under tillage and no tillage (Sun et al., 2018). Furthermore, the plowed soil could produce an even distribution of fungal species (Orrù et al., 2021). And the vertical rotation cultivation was helpful to improve the yield and profit of drought resistance of potato (Zhang et al., 2018). In addition, Behnke et al. (2018, 2021) revealed that the changes in tillage methods had the most direct impact on soil physicochemical properties, which have been reflected in previous studies that tillage methods could affect the soil environment by changing key soil properties, such as soil pH and organic matter (SOM).

In agricultural production, the changes in soil and microbial community structure of deep tillage have not been revealed, which may affect the yield and quality of tobacco to a certain extent. Therefore, in this study, a comparison experiment between HSs (deep-plowing treatments) and HCs (conventional tillage treatments) was conducted, and the physicochemical properties of soil and microbial diversity sequencing were used to compare the differences between HCs and HSs groups in different soil layers and microbial populations, so as to illustrate the improvement of soil microbial community by deep plowing. Heat map was used to analyze the interaction between environmental factors and microbial populations, which would provide new guidance for improving farmland management measures and optimizing activation modes of soil layer to further improve tobacco planting soil and increase crop yield.

## Materials and methods

### Description of study site

The selected test sites are located in Hefeng County, Enshi City, Hubei Province, China. They are located between longitudes 109°45′ and 110°38′ E and latitudes 29°38′ and 30°14′ N. The average annual temperature of the sampling site is 13–21°C, the average precipitation is 1,678 mm, and the soil type is yellow soil. The test site has been growing tobacco crops for two consecutive years. Two test fields were selected in this study, each with an area of about 200 m^2^.

### Experimental design and treatment

In this experiment, mature tobacco soils were studied. The growth period of tobacco is generally 120 days. The experimental group (HSs) used an excavator to plow at a depth of 40 cm and a ridge height of 30 cm. The control group was conventional tillage treatment (HCs), tobacco seedlings were planted normally, the soil layer activation depth was 20 cm, and the ridge height was 30 cm.

### Field procedure

The seedlings used in the two groups of tobacco seedlings were of the same quality and seedling age, and the land preparation, ridge raising, fertilization, film mulching, and transplanting were carried out at the same time, and the plant row spacing was 55 cm × 120 cm, and the fertilizer was uniformly applied according to the treatment. Good-growing tobacco plants are randomly selected. The control group and the experimental group applied the same amount of fertilizer, and the fertilization ratio was N:P_2_O_5_:K_2_O = 1:1.5:3, and the nitrogen application rate is 97.5 kg/hm^2^. After 10 days of transplanting smoke seedlings, potassium nitrate was applied.

### Soil sampling collection

Tobacco plants with good growth were randomly selected. We selected samples from two square plots (about 200 m^2^ in size) of deep-plowing and conventional tillage by five-point method, and randomly selected plants with good growth as test subjects (Zhang Y. et al., 2021). Five samples were collected from each soil depth, and a total of 40 samples were collected. The surface soil was scraped and collected with a sterile brush as a 0-cm soil layer sample. Then, soil samples with different tillage depths (HC1 vs. HS1: 0 cm, HC2 vs. HS2: 0–10 cm, HC3 vs. HS3: 10–20 cm, and HC4 vs. HS4: 20–40 cm) were collected along with the root of the plant with a soil drill, removed with a sterile brush and put into a sampling bag, transported to the laboratory in cold storage with ice bags, and stored at −80°C for testing.

### Detection of physicochemical properties of soil

Each sample was taken in triplicate at a time to ensure the uniformity of samples collected. The original soil samples were screened through an 18-mesh sieve (1 mm diameter) to avoid fine roots, plant residues, stones, and other stones. And the samples were stored in a refrigerator at −80°C for subsequent experimental analysis (Brow et al., 2010; Rissanen et al., 2010; Shen et al., 2021; Zhang Y. et al., 2021; Wang et al., 2022). After air drying, the basic physicochemical properties and enzyme activities of the soil were determined, including pH, water content (WC), total nitrogen (TN), soil organic matter (SOM), available phosphorus (AP), available potassium (AK), sucrase (S-SC), urease (UE), catalase (CAT), polyphenol oxidase (PPO), nitrate reductase (NR), and acid phosphatase (ACP) (Wang et al., 2019). Soil enzyme activities and soil physicochemical properties are measured as described in Supplementary File 1. In order to ensure the uniformity of sampling, all test samples were determined separately in triples. Testing the significance of ANOVA and diversity analysis was conducted on all soil enzyme activities and physicochemical properties to ensure the accuracy of the test, and a 95% confidence interval was set.

### DNA extraction and PCR amplification

The genomic DNA was extracted from the microbial community using the E.Z.N.A.^®^ SOIL DNA Kit (Omega Bio-Tek, Norcross, GA, United States). The DNA extract was determined by 1% agarose gel, and the concentration and purity of DNA were determined by NanoDrop 2000 UV-Vis spectrophotometer (Thermo Fisher Scientific, Wilmington, NC, United States). The highly variable region of bacterial 16S rRNA V3-V4 was amplified by using primers 338F (5′-ACTCCTACGGGAGCAGCAGC-3′) and 806R (5′-GGACTACHVGGGTWTCTAAT-3′) (Mori et al., 2014). PCR amplification of the 16S rRNA gene was performed as follows: initial pre-denaturation at 95°C for 3 min, followed by 27 cycles of denaturation at 95°C for 30 s, annealing at 55°C for 30 s, extension at 72°C for 45 s, and one extension at 72°C for 10 min and ending at 4°C. The PCR mixture consisted of 5 × TransStart FastPFU Buffer 4 μL, 2.5 mM DNTPs 2 μL, forward primer (5 μM) 0.8 μL, reverse primer (5 μM) 0.8 μL, TransStart FastPFU DNA polymerase 0.4 μL, template DNA 10 ng, and finally DDH_2_O reached 20 μL. The PCR reaction was repeated three times. PCR products were extracted from a 2% agarose gel and purified using the AxyPrep DNA gel extraction kit (Axygen Biosciences, Union City, CA, United States) and quantified using a Quantus™ fluorometer (Promega, San Luis Obispo, CA, United States) (Zhou et al., 1996; Cullen and Hirsch, 1998; Yeates et al., 1998; Zhao et al., 2005).

### Processing of sequencing data and bioinformatics analysis

The high-throughput sequencing was performed by the Shanghai Majorbio Bio-Pharm Technology Co., Ltd. (Shanghai, China). The raw 16S rRNA gene sequencing reads were demultiplexed, quality-filtered by fastp version 0.20.0 (Chen et al., 2018), and merged by FLASH version 1.2.7 (Mago and Salzberg, 2011) with the following criteria: (i) the 300 bp reads were truncated at any site receiving an average quality score of <20 over a 50-bp sliding window, and the truncated reads shorter than 50 bp were discarded, reads containing ambiguous characters were also discarded; (ii) only overlapping sequences longer than 10 bp were assembled according to their overlapped sequence. The maximum mismatch ratio of the overlap region is 0.2. Reads that could not be assembled were discarded; (iii) Samples were distinguished according to the barcode and primers, and the sequence direction was adjusted, exact barcode matching, two nucleotide mismatch in primer matching. After high-throughput sequencing, approximately 65,000 valid 16S rRNA gene sequences with an average length of 417 bp were obtained for each sample. The dilution curves of each sample were analyzed, which tended to be flat, indicating the saturation of operational taxonomic units (OTUs) and the validity of sequencing (Ye et al., 2017). Moreover, the coverage rates of all samples are close to 1, indicating that adequate coverage was enough (Chen et al., 2016). OTUs with 97% similarity cutoff were clustered using UPARSE version 7.1 (Stackebrandt and Goebel, 1994; Edgar, 2013), and chimeric sequences were identified and removed. The taxonomy of each OTU representative sequence was analyzed by RDP Classifier version 2.2 against the 16S rRNA database, using a confidence threshold of 0.7 (Wang et al., 2007).

The data used in this work are openly available in the NCBI Sequence Read Archive (SRA) at https://www.ncbi.nlm.nih.gov/bioproject/PRJNA730528 and the reference number is PRJNA730528.

### Data analysis

Two-way ANOVA statistical method in Prism software was used to test the significance of environmental factors (soil physicochemical and enzyme activity properties). The soil microbial data were analyzed using the free online program Majorbio Biocloud platform. Microbial diversity was evaluated by MOTHUR (Shanghai Majorbio Bio-Pharm Technology Co., Ltd.) and represented by Shannon index. Uparse (version7.0.1090) was used to perform OTU clustering analysis on the sequencing results, and the bacterial and archaea ribosomal data were compared in Silva database. Statistical tools of R language were used for Venn diagram analysis. Species significance tests were performed using R Stats and SciPy in Python to obtain genus levels with significant differences between groups. Principal component analysis (PCA) and redundancy analysis (RDA) were conducted by using R packages.

## Results

### Spatial differences in environmental factors after deep-plowing treatment

#### Changes in soil physicochemical properties

**FIGURE 1:**
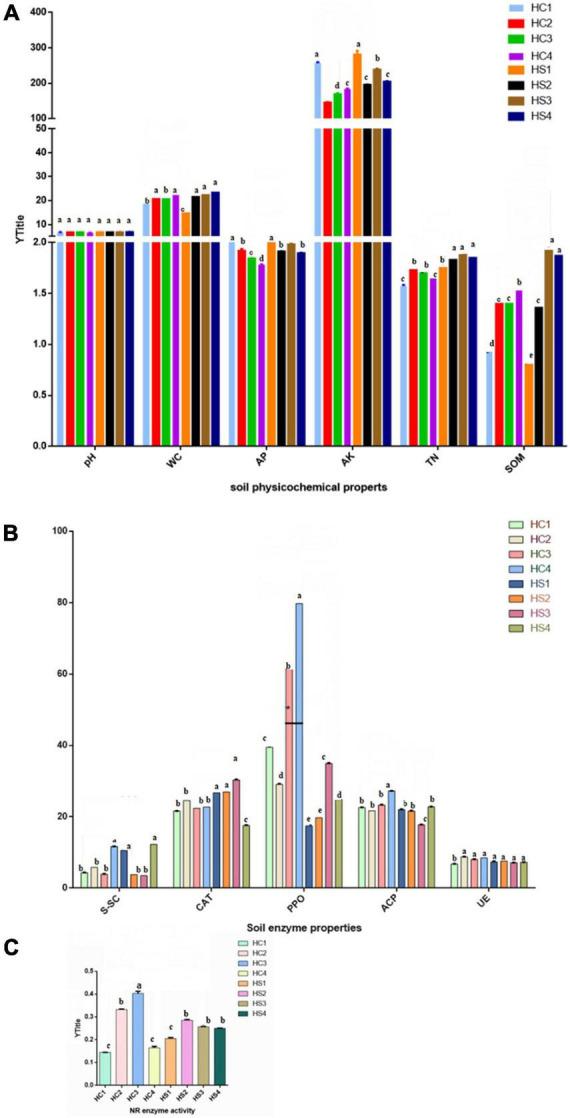
Physicochemical properties of soils in two groups. **(A)** Effects of deep tillage on soil pH, water content (%), available phosphorus (mg⋅kg^–1^), available potassium (mg⋅kg^–1^), total nitrogen (g⋅kg^–1^), and organic matter (g⋅kg^–1^); **(B)** Effects of deep tillage on soil sucrase (S), catalase (CAT), polyphenol oxidase (PPO), acid phosphatase (ACP), and urease (UE) activities. **(C)** Effect of deep tillage on nitrate reductase (NR) activity. Two-way ANOVA was adopted to examine the significance of the data. Mean values of treatments with the same letter are not significantly different in the figure; mean values of treatments where all letters were different are significantly different (*p* ≤ 0.05). Soil samples with different tillage depths (HC1 vs. HS1: 0 cm, HC2 vs. HS2: 0–10 cm, HC3 vs. HS3: 10–20 cm, and HC4 vs. HS4: 20–40 cm).

The physicochemical properties of soil foundation showed remarkable differences in vertical dimension in different soil layers of the two plowing treatments, including the deep-plowing group (HSs) and the conventional tillage group (HCs) (Figure 1). The pH of soil samples in the HCs group did not differ much at the soil level, as did in the HSs group. The moisture content (WC) of the HCs and HSs groups showed an increasing trend with the increase in soil longitudinal depth, and both reached the maximum value in the deep soil. Compared with the HC1 soil layer, the WC content of the HS1 soil layer has decreased. In addition, the results showed that the phosphorus nutrient content (AP) of the HCs sample decreased significantly with the increase in soil depth. In the HSs group, the AP content changed with soil depth, and the content was higher in HS1 and HS3 and lower in HS2 and HS4.

The AK content in the HCs group increased first along with the longitudinal gradient and then decreased, and the AK content in the HSs group showed a decreasing–rising–decreasing trend. It was also found that the AK content of the HSs group was higher in the same soil layer than in the HCs group. In addition, both groups of soil TN decreased with soil depth. However, the difference is that the TN content of the HCs group gradually decreased from the 0–10 cm soil layer sample (HC2), that is, the 10–20 cm soil layer sample (HS3) in the HSs group. Moreover, SOM levels increased in the HCs group, but it did not change much in the two layers below the surface (HC2 and HC3). Interestingly, the SOM content of each soil layer increased significantly with depth after deep plowing, and HS3 reached a maximum of 1.92 g/kg.

#### Changes in soil enzyme activities

Soil enzyme activity at longitudinal depth after deep plowing (HSs) was inconsistent with the HC sample (Figures 1B,C). Among the HCs group, the soil sucrase (S-SC) activity was the strongest in the HC4 soil layer, and it showed obvious differences with the HC1, HC2, and HC3 soil layers. In the HSs group, the enzyme activity of S-SC was not significantly different in the HS1 and HS4 soil layers, and compared with the HCs group, S-SC showed a decreasing–rising trend with the increase in soil depth. At the same time, the results showed that the difference in soil CAT activity between the soil layer samples in the HCs group was not significant, while the enzyme activity of the HS4 samples decreased significantly between the soil layer samples in the HSs group. Furthermore, the results showed that the soil PPO activity trends were also different in the two groups. PPO decreased first and then increased as the soil depth increased in HCs groups. However, HC1 and HC2 were not significantly different between the HSs groups, and with the increase in longitudinal soil depth, the enzyme activity showed a trend of first increasing and then decreasing. In addition, the PPO of the same soil layer in the HSs group was lower than that in the HCs group. The ACP activity of the HC4 samples was significantly higher than that of the HC1, HC2, and HC3 samples, and ACP increased along with the longitudinal depth. After deep tillage, the ACP of the HS3 samples was significantly lower than that of HC1, HC2, and HC4 and higher with the longitudinal depth. UE activity in both sets of samples did not change significantly along with the longitudinal depth. The NR activity of the HCs group was first raised and then decreased along with the soil depth and showed significant differences in various soil layers. Interestingly, the differentiation among HS2, HS3, and HS4 has weakened after deep tillage (Figure 1C).

### Changes in microbial community structures after deep-plowing treatment

**FIGURE 2:**
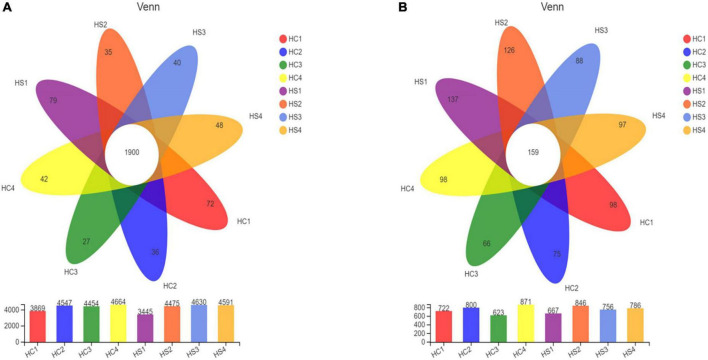
Venn analysis shows common and endemic species among soil samples [panel **(A)** for bacteria, panel **(B)** for fungi].

In order to determine the overall composition of the microbial community, a Venn diagram of the sample community [based on the statistics and mapping tools of the software: R language (version 3.3.1)] was produced. After deep-plowing treatment, the number of bacteria-specific OTUs in the samples increased between each soil layer. There were 1,900 bacterial OTUs shared in all samples. The unique OTUs of HC1, HC2, HC3, and HC4 were 72, 36, 27, and 42, respectively, and the unique OTUs of HS1, HS2, HS3, and HS4 were 79, 35, 40, and 48, respectively (Figure 2A). Similarly, 159 fungal OTUs were shared in the samples, and the number of unique OTUs in the 0-cm layer in the two groups was also the highest (Figure 2B). Obviously, the number of OTUs in both of the two groups showed a similar trend with the increase of soil depth. It is worth noting that the number of OTUs of HS3 was significantly increased compared with that of HC3, and the number of OTUs of HS1, HS2, and HS3 was also significantly increased compared with that of HC samples. These results indicated that deep tillage treatment raised a great impact on soil microbial community and structure.

To reveal the effects of deep tillage on soil microorganisms, the richness and diversity of soil microbial communities between different soil layers were analyzed by Alpha Diversity Analysis (Table 1). The results showed that the cover index of each sample is close to 1, indicating that the sample score coverage is sufficient. The results of the analysis of bacterial α diversity were as follows, HS1 (323) < HC1 (336.33), HS2 (364.33) < HC2 (365.33), HS3 (369.00) > HC3 (357.00), and HS4 (367.00) < HS4 (367.67) across samples, indicating that deep tillage affected microbial community richness. Shannon index results were HC1 (4.11) > HS1 (4.06), HC2 (4.37) < HS2 (4.39), HC3 (4.38) < HS3 (4.49), and HC4 (4.40) < HS4 (4.44). Deep tillage affected microbial community diversity in deep soils. The Heip index showed almost no change in the two groups of samples, indicating that the degree of community uniformity was almost unchanged. These results indicated that the richness and diversity of tobacco microbial community varied significantly in soil at different depths, which are consistent with the previous study (Sun et al., 2018).

**TABLE 1:** Alpha diversity analysis (A, bacteria; B, fungi) of two treatment groups.

Sample	Sobs	Shannon	Coverage (%)	Heip
A				
HC1	336.33 ± 14.36^a^	4.11 ± 0.16^a^	99.94 ± 0.00^a^	0.18 ± 0.02^a^
HC2	365.33 ± 3.51^a^	4.37 ± 0.04^a^	99.91 ± 0.00^a^	0.22 ± 0.01^a^
HC3	357.00 ± 2.65^a^	4.38 ± 0.05^a^	99.90 ± 0.00^a^	0.22 ± 0.01^a^
HC4	367.67 ± 4.73^a^	4.40 ± 0.05^a^	99.93 ± 0.00^a^	0.22 ± 0.01^a^
HS1	323.67 ± 7.02^a^	4.06 ± 0.03^a^	99.92 ± 0.00^a^	0.18 ± 0.00^a^
HS2	364.33 ± 0.58^a^	4.39 ± 0.02^a^	99.92 ± 0.00^a^	0.22 ± 0.00^a^
HS3	369.00 ± 11.36^a^	4.49 ± 0.06^a^	99.92 ± 0.00^a^	0.24 ± 0.01^a^
HS4	367.00 ± 5.29^a^	4.44 ± 0.05^a^	99.93 ± 0.00^a^	0.23 ± 0.01^a^
B				
HC1	103.67 ± 11.68^a^	2.38 ± 0.31^a^	99.99 ± 0.00^a^	0.10 ± 0.04^a^
HC2	120.33 ± 2.31^a^	2.12 ± 0.06^a^	99.99 ± 0.00^a^	0.06 ± 0.00^a^
HC3	118.33 ± 5.77^a^	1.93 ± 0.10^a^	99.99 ± 0.00^a^	0.05 ± 0.00^a^
HC4	114.67 ± 8.50^a^	1.98 ± 0.15^a^	99.99 ± 0.00^a^	0.06 ± 0.01^a^
HS1	97.33 ± 8.74^a^	2.36 ± 0.15^a^	99.99 ± 0.00^a^	0.10 ± 0.01^a^
HS2	122.33 ± 5.03^a^	2.07 ± 0.29^a^	99.99 ± 0.00^a^	0.06 ± 0.02^a^
HS3	115.00 ± 9.00^a^	2.16 ± 0.16^a^	99.99 ± 0.00^a^	0.07 ± 0.01^a^
HS4	112.67 ± 3.06^a^	2.16 ± 0.08^a^	99.99 ± 0.00^a^	0.07 ± 0.01^a^

Shannon, Simpson, Ace, Chao, and coverage are commonly used metrics to reflect community richness and diversity. Results are expressed as mean value ± standard error.

Mean values of treatments with the same letter are not significantly different in the figure; mean values of treatments where all letters were different are significantly different (p ≤ 0.05). Soil samples with different tillage depths (HC1 vs. HS1: 0 cm, HC2 vs. HS2: 0–10 cm, HC3 vs. HS3: 10–20 cm, and HC4 vs. HS4: 20–40 cm).

#### Changes in bacterial communities

**FIGURE 3:**
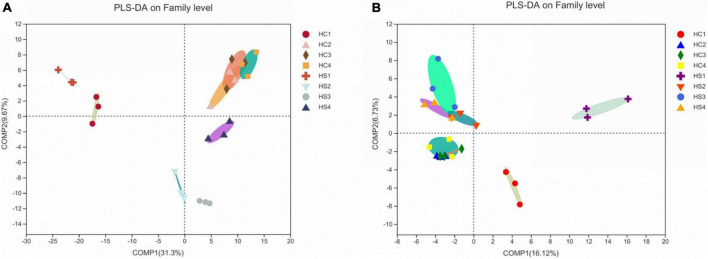
PLS-DA analysis in sample microbial community. **(A)** Analysis of bacterial communities at the family level; **(B)** Analysis of fungi communities at the family level.

**FIGURE 4:**
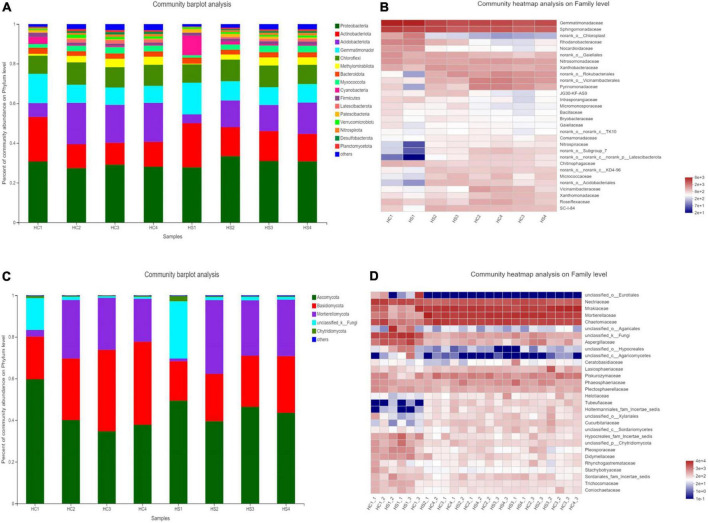
Composition and structure of the microbial community in soil samples from different soil layers in phylum level classification [panel **(A)** for bacteria; panel **(B)** for fungi]. Heatmap of microbial community abundance in family level classification [panel **(C)** for bacteria; panel **(D)** for fungi].

**FIGURE 5:**
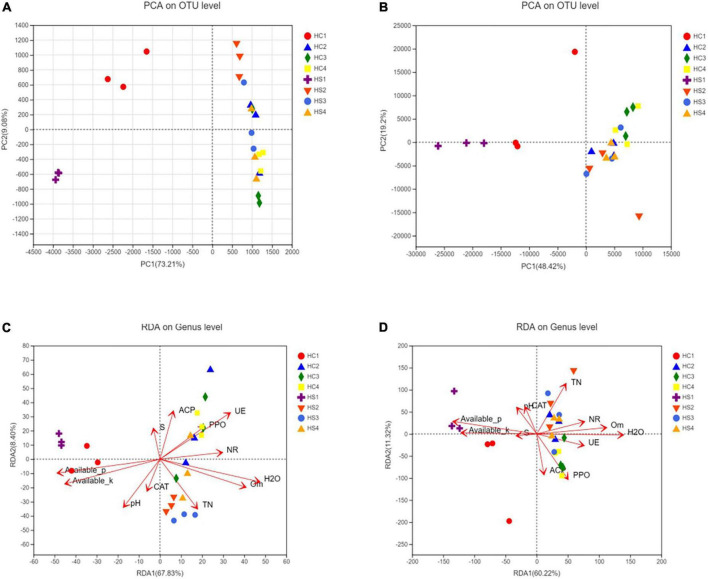
Principal component analysis (PCA) analysis of bacterial and fungal **(A,B)** communities in a β-diversity analysis of tobacco soil microbial community; RDA analysis between bacterial or fungal community and environmental factors, respectively **(C,D)**.

It is obvious that the composition and structure of bacterial community varied after deep plowing. Bacterial community composition of HC2, HC3, HC4, and HS2, HS3, and HS4 showed significant differences in the COMP2 axis. The differences among samples fully explained the differences between soil microbial community structural diversity after deep tillage and conventional tillage. Meanwhile, on the COMP1 axis, the microbial structure of surface soil samples HC1 and HS1 was significantly different from that of deep soil samples HC2, HC3, HC4, and HS2, HS3, and HS4 (Figure 3A). To verify whether bacterial communities are different, PCA analysis of bacterial community structures in individual samples at the OTU level using R packages was performed (Figure 5A). The results showed that the interpretation of the PC1 and PC2 axes was 73.21 and 9.08%, respectively, and there were significant differences between the soil samples after deep plowing and the control group, indicating that the bacterial community structure changed greatly after deep plowing. At the phylum level (Figure 4A), Proteobacteria, Actinobacteria, Acidobacteria, Blastomonas, and Chlorobacteria with different proportions were dominant in all the samples, among which Proteobacteria and Actinobacteria were predominant, especially in HC1 and HS1 samples. In HC3, the top four dominant phyla were Proteobacteria (29.15%), Actinobacteria (11%), Acidobacteria (19.12%), and Blastomonas (8.77%), the same as those in HS3 samples, with different proportions of 30.94, 15.14, 13.13, and 8.97%, respectively. This implied that deep-plowing led to an increase in the community diversity of Proteobacteria, Actinobacteria, and Blastomonas. However, Acidobacteria seemed to be inadaptable to the changes after deep plowing and presented a downward trend. In other soil layers, different changes in the flora were also found. Compared with the 0-cm samples (HC1 and HS1), the proportion of Acidobacteria in other samples (HC2, HC3, HC4, HS2, HS3, and HS4) increased significantly, along with the obvious decrease in the proportions of Actinobacteria and Blastomonas. In addition, the dominant phyla contained in the deep-plowing treated samples showed high abundance, including Proteobacteria, Actinobacteria, Acidobacteria, Blastomonas, Chlorophyta, Methylomirabilota, Plancobacteria, Bacteroidetes, Firmicutes, and Cyanobacteria. The relative abundance of Methylomirabilota in HC1 samples is lower than HC2, HC3, and HC4. Interestingly, the abundance of Methylomirabilota increases gradually as the soil depth increases. In HC3 sample, the richness of this phylum reached to the highest level, and the abundance of this phylum hardly changed in HC3 and HC4 samples, which were consistent with the results of previous studies (Zhang X. et al., 2021). The change mode of Methylomirabilota in HS group showed the same trend, indicating that soil disturbance has little effect on this phylum in the bacterial community. Moreover, the abundance of cyanobacteria in different soil layers decreased in HS group along with the increase in soil depth, showing a significant negative correlation. In HC group, HC1 revealed the highest richness, while HC2, HC3, and HC4 showed no significant changes. The richness of HS1 and HS2 was higher than that of the corresponding HC1 and HC2, which might be attributed to the increase in the cyanobacteria abundance in soil samples after plowing treatment. As shown in Figure 4B, soil microbes were further subdivided to the level of families. It was observed that in the two groups, almost all bacteria presented high abundance at the family level. In HS1 and HC1 samples, only a small amount of bacteria, such as Norank-O-Acidobacteriales, had a low abundance.

#### Changes in fungal communities

The fungal community structure also changed somewhat after deep tillage (Figure 3B). The COMP1 axis explained the differences in samples by 16.12%. The fungal community structure of the surface soil samples (HC1 and HS2) and the deep soil samples (HC2, HC3, HC4, HS2, HS3, and HS4) were different, indicating the differentiation of the fungal community structure between the topsoil and the deep soil. The COMP2 axis had a 6.73% degree of interpretation for the sample differences, and there were significant differences in fungal communities in the HCs group and HSs groups on this axis, indicating that there were obvious differences in fungal community structure between the treatment group and the control group. Similarly, PCA analysis at the sample fungal OTU levels was performed (Figure 5B). The results showed that deep tillage had raised less influence on fungal communities. The dominant fungal phylum in the samples was Ascomycota, Basidiomycota, Mortierellomycota, unclassified_k_fungi, and Chytridiomycota, whose proportions in the 0 cm samples (HC1 and HS1) were significantly different from that in other samples (HC2, HC3, HC4, HS2, HS3, and HS4), especially for Mortierellomycota and unclassified_k_fungi (Figure 4C). These shared fungi were affiliated to the phyla generally found in soils with personalized functions. In the soil samples below 0 cm (HS2, HS3, HS4, HC2, HC3, and HC4), the abundance of Mortierellomycota after deep plowing was significantly improved, which was of great help to the release of organic substances in the soil. Cluster analysis was performed on the heatmap of fungi community at a family level, and the species with abundance ranked in the top 30 were displayed (Figure 4D). Mortierellaceae, Nectriaceae, Chaetomiaceae, and other families were found to have a large number of common microorganisms in the deep soil. Morphomycetes are a class of saprophytic fungi, which are abundant in organic matter-rich soils, indicating that the content of SOM in deep soil samples is higher than that in surface soil samples. This is consistent with the gradual increase in SOM content in vertical depth as described above. Moreover, the number of fungal OTUs was smaller than that of bacterial ones. Meanwhile, the number of fungal phyla with great proportion was also much smaller than that of bacterial phyla.

### Correlation analysis between soil physicochemical properties and microbial diversity

Beta diversity analysis based on Bray–Curtis distance showed that the bacterial community structure in the HS group was significantly changed compared to that in the HC group (Figures 5A,B). The OTU cycle of the samples varied greatly after deep-plowing treatment, indicating that deep-plowing affected the bacterial microbial diversity in different soil layers. The three replicates showed good repeatability in every sample, and the topsoil samples (HC1 and HS1) were distributed far away. On the contrary, the relative difference between deep soil samples was smaller.

Environmental factors interacted significantly with bacterial community diversity (Figure 5C). The deep-plowing treatment altered the main environmental factors that contribute to the diversity of stratified bacterial communities in soil. Furthermore, some of the environmental factors, including WC, AP, AK, SOM, UE, NR, and PPO, were closely related to the differences in microbial community structure, and WC, AP, AK, SOM, UE, and other environmental factors were extremely significantly correlated with the difference in community structure. NR and PPO were significantly correlated with the difference in sample bacterial community, while pH, TN, ACP, CAT, and S-SC were not significantly correlated with the difference in sample bacterial community (Supplementary Table 1). As the first major environmental factor affecting soil stratified diversity, the degree of influence of pH on bacterial community diversity increased after deep plowing (Figure 5C). Inversely, there was no significant correlation between bacterial diversity and pH in HC samples. Interestingly, although the correlations between the basic soil physicochemical properties and bacterial diversity after deep-plowing treatment have changed, the interactions between enzyme activities and bacterial diversity were more significant. In the HC group, HC2, HC3, and HC4 showed a remarkable positive correlation with the contents of WC, TN, and SOM in the soil. However, HS2, HS3, and HS4 samples showed a significant positive correlation with the enzyme activities of ACP, PPO, S-SC, UE, and NR, and their microbial diversity increased with the increase in these enzyme activities. Moreover, the enzyme activities of ACP, PPO, and UE showed more significant interactions with bacterial diversity. In the same group, the relevance degree of different environmental factors with bacterial diversity in each soil layer is not consistent. Among all environmental factors, the contents of AP and AK mainly interacted with soil bacterial diversity at 0 cm surface, which was more obvious in the deep-plowing samples. And soil AP and AK were mainly negatively correlated with the soil samples below 0 cm. All these results indicated that the deep-plowing treatment raised great influences on the correlations between environmental factors and bacteria community diversity along with the soil depth. Similarly, environmental factors also showed significant correlations with the diversity of fungal communities (Figure 5D). In HC group, AK was the main environmental factor, showing a positive correlation with HC1, and the enzyme activity of S-SC hardly interacted with the fungal diversity of samples. HC2 sample presented a positive correlation with WC, SOM, and NR, and HC3 and HC4 showed a positive correlation with PPO and ACP. Comparatively, HS1 had a stronger positive correlation with AP, and HS2 showed a stronger positive correlation with TN. Moreover, HS3 and HS4 demonstrated a strong positive correlation with WC, TN, SOM, and NR. In general, the deep-plowing treatment has also disturbed the correlations between environmental factors and fungal community diversity in different soil layers.

**FIGURE 6:**
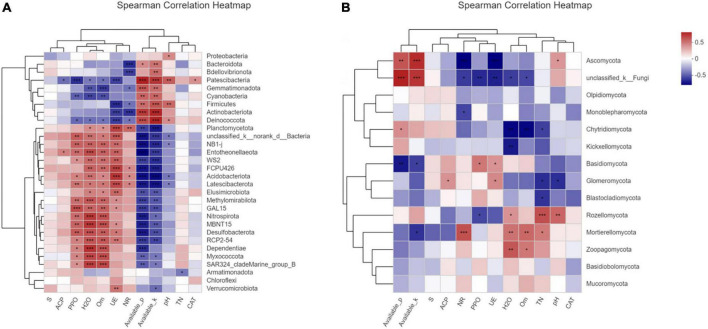
Correlation heat map was used to analyze the correlation between environmental factors and communities at the phylum level of bacteria **(A)** or fungi **(B)** in soil samples, so as to represent the correlation coefficient between each microorganism in the community and environmental factors, thus indicating the relationship between microbial classification and environmental factors. The asterisk indicates a statistically significant *t*-test analysis. *, **, and *** represent *p*-values ≤ 0.05, 0.01, and 0.001, respectively.

Compared with fungi phyla, the bacteria phyla had a more regular correlation with environmental factors as in the correlation heatmap analysis (Figures 6A,B). S-SC, ACP, CAT, and TN showed no significant correlation with the dominated bacterial phyla. However, UE, NR, and PPO presented a strong correlation with this phyla. There was a significant negative correlation between the abundance of Bacteriadota, Bdellovibrionota, Patescibacterium, Blastomonas, Gemmatimonadota, Cyanobacteria, Firmicutes, Actinobacteriota, Deinococcota, and the environmental factors including PPO, UE, and NR, in contrast to a significant positive correlation between the abundance of Deinococcota and AP, AK. Proteus is sensitive to soil pH, and other environmental factors have little correlation with it. Two enzymes, UE and PPO, promoted the activity of the enzyme Planctomycetota, ND1-J, WS2, FCPU426, Acidobacteriota, Latescibacterota, GAL15, Nitrospirota, Desulfobacterota, Dependentiae, and other bacteria abundance increased. Similarly, the increase in organic matter (SOM) and WC also promoted these bacteria. In contrast, the presence of AK and AP reduced the abundance of these bacteria. Obviously, the fungal phyla were less correlated with environmental factors than bacterial phyla (Figure 6B). Unclassified K-fungi were highly correlated with AP and AK, and significantly negatively correlated with PPO and UE. Meanwhile, it is negatively correlated with NR, WC, and SOM. Kichxellomycota and chytrid fungi were negatively correlated with WC. In summary, the relationship between fungal communities and environmental factors is irregular and not obvious, indicating that the effects of fungi on soil enzyme activity and nutrient transformation are diversified (Figure 6B). The relationship between fungal communities is generally less clear than that of bacterial communities, so the function of fungal communities in plant soils needs to be studied further.

## Discussion

### Environmental factors in spatial dimension changed significantly

As reported, environmental factors could affect tobacco growth, and different environmental factors showed different influences on plant growth (Tsadilas, 2000; Li et al., 2011). In this work, it can be seen that these environmental factors varied greatly between different soil samples, but some are relatively stable. Generally, the root depth of tobacco was about 15–20 cm underground. Obviously, the change in HS3 (10–20 cm underground layer) has a great influence on tobacco growth. The increase in SOM and CAT in HS3 samples after deep tillage was obvious. It was speculated that deep plowing may have disturbed the microbial structure of the soil, while tobacco root growth can take root at depths of 15–20 cm underground, and HS3 samples and HC3 samples are in 10–20 cm soil layers. The microbial diversity of the HS3 sample was stronger than that of the HC3 sample, which indirectly indicated that the soil biomass increased and the increase in plant productivity led to an increase in the absolute amount of litter. This alteration may be related to the action of tobacco root secretions, which may be the reason why HS3 differs from HC3 SOM. Previous reports have stated that soils with 3% organic matter content and high CAT activity were considered suitable for tobacco growth (Kizilkaya and Ekberli, 2008; Ye et al., 2010), indicating that the increase in the SOM content and CAT activity plays an extremely important role in the growth of tobacco. In addition to these two environmental factors, the contents of pH, TN, water (WC), AP, and AK in HS3 samples were all higher than those of HC3 samples after deep plowing. As known, sufficient soil capillary water and available nutrient accumulation could promote the growth of plants. Therefore, it was deduced that the increase in soil permeability after deep plowing has improved root respiration and changed soil microbial diversity, which may promote the change in soil physicochemical properties. Furthermore, the activity of partial enzymes was inhibited after deep tillage. Soil sucrase (S-SC), UE, and NR were secreted to soil by plant roots and soil microorganisms, which could promote the transformation of some substances in the soil within the range of the root system, being conducive to the uptake and utilization of rhizosphere microorganisms. The activity of ACP was also weakened, which directly affected the decomposition and transformation of organic phosphorus in soil as the same as its bioavailability. Deep tillage reorganized soil microbial community structure, and it took some time for the corresponding microorganisms to establish a new rhizosphere effect, which may be the reason for the inhibition of some enzyme activities, such as PPO.

In soil samples at different depths, the physicochemical properties of each sample also showed differences and specificity, which was consistent with the previous report (Liu et al., 2017). It has ever been reported that soil UE was positively correlated with the contents of SOM and TN (Reynolds et al., 1985). After deep tillage in this work, the content of organic matter was significantly increased; however, UE and NR were evenly distributed in all soil layers, which had weakened the differences in the dynamic changes in enzyme activities in spatial distribution. Similarly, CAT activity has not varied with the increase in soil depth. The weakened dynamic changes in enzyme activities indicated that deep tillage had raised a significant effect on the biochemical characteristics of cultivated plants, which may be one of the reasons for the change in environmental factors.

### The microbial diversity varied dynamically

In general, the diversity indices of the deep-plowing treated soil samples (HS1, HS2, HS3, and HS4) were all higher than those of the controlled samples (HC1, HC2, HC3, and HC4), and the differences were most obvious in the soil with 10–20 cm depth. The higher Sobs index of HS3 compared with that of HC3 demonstrated that the abundance of HS3 community was improved after deep tillage. HS1 sample presented the lowest community diversity with the lowest Shannon index. The obvious case for the change is that deep tillage has significantly affected soil physicochemical properties, following resulted in the change in microbial diversity. As a result, the bacterial species included in HS3 were different from those in HC3, and the number of specific strains in HS3 was also higher than that in HC3, both of which might be also due to the changed soil environmental factors after deep tillage. Based on the comparison of HC3 and HS3, it could be found that deep tillage resulted in the deformation of Actinobacteria and the increase in Bacillus community diversity, and Actinobacillus did not seem to adapt to the plowing treatment. It was suspected that this may be due to the increase in the soil permeability, and aerobic bacteria had grown quickly. The increase in aerobic bacteria may compete for resources for the growth of some bacteria, which may be the reason for the decrease of Acidobacteria, Actinomyces, and Spomonas, which are mostly aerobic bacteria.

As the analysis of beta diversity, it was inferred that different soil physicochemical properties would lead to great differences in bacterial communities. In the soil samples, Proteobacteria accounted for the largest proportion of all the bacterial communities. As reported, the communities formed by Proteobacteria played an important role in nitrogen circulation in the soil (Grün and Emmerling, 2018). Meanwhile, cyanobacteria have special ways to control nitrogen in their bodies, which could help release nitrogen from the soil (Mian and Stewart, 1985). This might be the reason why plowing can increase soil nitrogen content, which was verified by the result that the TN content of the soil samples after plowing was higher than that of the unplowed soil, particularly obvious in the soil samples with 10–20 cm depth. This may be due to the increase in proteobacteria after plowing.

Actinobacteria is the second largest group of bacterial species in the soil samples. Most of the bacteria in this phylum can produce indole acetic acid and ammonia, which can also fix N_2_, all these properties of actinobacteria would promote tobacco growth (Laassami et al., 2020). For the fungal community, unclassified_k_fungi with strong cellulose dissolution ability was present in the samples (Song et al., 2020). As mentioned above, these shared microorganisms in the samples, such as Mortierellomycota, have been reported as effective organic matter transformers (Santillán et al., 2021). As a result, the abundance of Mortierellomycota in the samples after plowing has been significantly improved, which was of great help to the release of organic substances in the soil. After deep tillage treatment on HS3, the abundance of sporophyte was significantly increased, and it could also greatly help the release of organic matter in the soil. Morphological fungi are saprophytic fungi, which are abundant in soil rich in organic matter, indicating that the content of organic matter in deep soil samples was higher than that in surface soil samples. This was consistent with a gradual increase in SOM along with the vertical depths. As reported, the vertical change in microbial community was correlated with the decomposition degree of SOM (Kim et al., 2016). Although the abundance of bacterial diversity in soil samples is much greater than that of fungi, the role played by fungi cannot be ignored to some extent. Fungi still played a certain role in improving soil enzyme activity and maintaining microbial community structure (Nacke et al., 2011). For example, some fungi can have a positive effect on tobacco photosynthesis by forming mycorrhiza with plant roots, which also participate in root nutrient uptake (Kamińska et al., 2010).

### Microbial community interacted with environmental factors showed obviously

After deep tillage, the soil environmental factors changed significantly and the microbial community structure in the corresponding ecological niche also varied a lot, during which course the role of rhizosphere microorganisms should not be ignored. Soil microorganisms can directly or indirectly participate in soil structure improvement, nutrient utilization, and disease occurrence. Moreover, it has been reported that the soil microbial community is the core driving force of nutrient cycling in cultivated soil, including improving soil physicochemical properties and interacting with soil enzyme activities (Khare and Arora, 2015; Celestina et al., 2019). Deep tillage had brought about changes in soil physicochemical properties and enzyme activities, and rhizosphere microorganisms responded to soil nutrients and enzyme activities, adapting to the new soil environment with new community composition. Therefore, environmental factors have important effects on the microbial community and the growth process of tobacco.

Entotheonellaeota, Dependdiatiae, and Nitrospirota showed a high correlation with SOM (Figure 6A). It has been reported that these three types of bacteria are enriched during the mature stage of compost fermentation (Araujo et al., 2021). The increase in organic matter content after deep tillage may result from the increase in the abundance of these three bacteria. Although pH is not strongly correlated with the abundance of most bacteria, the bacterial diversity of samples after deep tillage had also increased with the increase in pH value, which might be deduced that deep tillage affected the change in pH, and following lead to the variation of soil bacterial community structure. Moreover, the relative abundance of Acidobacteria was significantly positively correlated with soil organic carbon, which was consistent with the previous report (Fierer et al., 2007; Naether et al., 2012). As reported, plant root exudates could affect soil enzyme activities, thus affecting soil microbial diversity, which was also verified by the experimental results. Bacillus is usually particularly resistant to adverse environmental conditions, and many of them were rhizosphere bacteria that are conducive to plant growth. In the newly formed microbial community structure after deep tillage, the presence of a large number of Bacillus would be of great help to maintaining the activities of PPO and CAT. In addition, Bacillus can secrete a variety of bioactive substances, such as antibacterial proteins and growth hormones, to improve crop resistance, inhibit the occurrence of disease, and promote crop growth and development (Yánez-Mendizábal and Falconí, 2018; Anusha et al., 2019). According to the results, the decrease in UE may be closely related to the newly formed dominant flora structure, which could effectively reduce UE and promote the slow release of nitrogen (Subraman and Kannayaan, 1989; Lawler et al., 2013). This may also contribute to the increase in soil TN content after deep tillage. Furthermore, the percentage of actinomycetes was significantly increased in the soil microbial community after deep tillage, which played an important role in organic matter circulation. The rhizosphere could inhibit the growth of a variety of plant pathogens; decompose complex polymer mixtures in plant, animal, and fungal materials; and produce a variety of extracellular enzymes that contribute to crop production. Additionally, actinomycetes play a significant part in soil bio-buffering, biological control of soil environment by nitrogen fixation, and degradation of high molecular weight compounds, such as hydrocarbons, in contaminated soil. In short, actinomycetes could improve the availability of nutrients, minerals, metabolite production, and plant growth regulators (Bhatti et al., 2017). Therefore, the soil microbial structure including increased actinomycetes abundance after deep tillage would be more conducive to the growth of tobacco plants.

The abundance and species differences of dominant fungi were not significant in this study; however, this did not mean fungi were not important in the soil environment as mentioned above. According to the correlation analysis between fungal groups and soil environmental factors, PPO, CAT, and TN had presented significant interactions with Sordariales, Chaetothyriales, Hynodontacae, Tremellales, Pseudomycetes, and Agaricales. According to previous studies, the relationship between fungal communities and environmental factors is irregular. The effects of fungi on soil enzyme activity and nutrient transformation are unclear and need to be further revealed in future work.

## Conclusion

In conclusion, as an agricultural land management practice, this work suggested that deep tillage could cause a strong disturbance to the biological and physicochemical properties of the soil. Deep tillage has affected the microbial community structure, along with the changes in environmental factors, such as SOM and CAT, which were conducive to plant growth and development. Meanwhile, the variation of soil environmental factors interacted with the difference in soil microbial community composition and structure. With the increase in soil depth, the diversity of community structure changed significantly, which was related to the changes in soil physicochemical properties and enzyme activities. The increased diversity of rhizosphere bacteria has laid the foundation for the formation of stable rhizosphere. These results together indicated the spatial differences in microbial diversity and environmental factors after deep tillage. In contrast to previous studies, this work would propose a new field management practice, that is a protective depth of 40 cm tillage. In brief, deep tillage improved the soil environment, increased the abundance of beneficial microorganisms, and affected soil biota, which may have implications for soil sustainability and agricultural parameters. Moreover, the dynamic interaction between environmental factors and microbial community played an important role in the growth and development of tobacco, which presented important guiding significance for studying the interaction system between plant, soil, and microbial, and improving crop yield and quality.

## Data availability statement

The datasets presented in this study can be found in online repositories. The names of the repository/repositories and accession number(s) can be found below: https://www.ncbi.nlm.nih.gov/, PRJNA730528.

## Author contributions

ZW and ZS conceived the idea for the study. ZW, GS, JY, and SD designed the experiment work. YZ and GB collected and analyzed the data. YZ, GB, and ZW wrote the manuscript. MS helped to revise the manuscript. YZ revised the manuscript after reviewing it. All authors have read and approved the manuscript and reviewed the manuscript.

## Supplementary material

Supplementary Table 1 Permutational multivariate analysis of variance.

Supplementary File 1 Methods of enzyme activity detection.
