# Supplementary material for: Differences in microbial diversity and environmental factors in ploughing-treated tobacco soil
Source: Front Microbiol. 2022 Sep 12;13:924137. doi: 10.3389/fmicb.2022.924137 (PMC9511222; doi:10.3389/fmicb.2022.924137)
Supplement: Supplementary Table 1 — Permutational multivariate analysis of variance. [file Table_1.pdf]

Supplementary Table 1. Permutational Multivariate Analysis of Variance

Characteristics	F.Model	R^2	P.value	P.adjust
WC	26.49188	0.54632	0.001	0.003
AP	29.84335	0.57564	0.001	0.003
Ak	29.84335	0.57564	0.001	0.003
SOM	14.25702	0.39322	0.001	0.003
UE	8.40327	0.27639	0.002	0.0048
NR	5.02234	0.18586	0.007	0.014
PPO	3.52909	0.13824	0.025	0.04286
pH	2.42942	0.09945	0.081	0.112
TN	2.57698	0.10485	0.084	0.112
ACP	1.3272	0.05689	0.231	0.2772
CAT	1.04178	0.04521	0.313	0.34145
S-SC	0.47631	0.02119	0.63	0.63
