# Supplementary material for: Differences in microbial diversity and environmental factors in ploughing-treated tobacco soil
Source: Front Microbiol. 2022 Sep 12;13:924137. doi: 10.3389/fmicb.2022.924137 (PMC9511222; doi:10.3389/fmicb.2022.924137)
Supplement: Supplementary File 1 — Methods of enzyme activity detection. [file Data_Sheet_1.docx]

# Supplemental File 1. Methods of enzyme activity and soil physicochemical property detection.

**Methods of enzyme activity detection**

The activities of soil urease, acid phosphatase and nitrate reductase were detected using Soil Urease (S-UE) Assay Kit (BC0120, Solarbio Co., China), Soil Acid Phosphatase (S-ACP) Assay Kit (BC0140, Solarbio Co., China) and Soil Nitrate Reductase (S-NR) Assay Kit (BC3100, Solarbio Co., China), respectively. And the detailed methods for detecting the activities of soil polyphenol oxidase, catalase and sucrase were listed below.

# Detection of soil polyphenol oxidase

Soil polyphenol oxidase was determined by ultraviolet spectrophotometry. The polyphenol oxidase activity was indicated by the amount of gallin which was extracted with ethyl ether and determined by ultraviolet spectrometric colorimetry.

The specific steps are as follows: 1 g of the air-dried soil sample passed through a 1mm sieve was placed into a 50 mL centrifuge tube, and then 10 mL of 1% pyrethol solution was injected without a cover. The centrifuge tube was placed in a constant temperature vibration incubator for constant temperature culture at 30℃ for 2h. After extraction, 4 mL citric acid-phosphoric acid buffer with pH value of 4.5 was added, and 10 mL ethyl acetate was added for extraction for 30min. The pigmented ether (ethyl acetate) phase extracted liquid containing red purple gallin was colorimetric at 430 nm and compared with the standard curve of red purple gallin. In order to eliminate the deviation caused by the original ether-soluble organic matter in the soil, the soil - free and matrix - free controls were made simultaneously.

# Detection of soil catalase

The activity of soil catalase was determined by ultraviolet spectrophotometry. Catalase promotes the enzymatic breakdown of hydrogen peroxide to produce water and oxygen. By adding excessive amount of hydrogen peroxide and interacting with soil for a period of time, the difference between the added amount and the remaining amount is the hydrogen peroxide consumed by the enzyme-catalyzed reaction, which represents the enzyme activity.

The specific steps are as follows: place 0.5 g of the soil sample into a large centrifuge tube, add 9 mL distilled water and 4 mL 0.3% H2O2 solution, and leave it in the dark place for 20 minutes. Remove and quickly add 1 mL of saturated aluminosalum, and filter into a triangle flask containing 1 mL of 1.5 mol/L sulfuric acid solution. The absorbance As was measured at 240 nm with a 1 cm quartz colorimetric dish. Soilless A0 and matrix - free AK were compared simultaneously.

# Detection of soil sucrase

The soil sucrase activity was determined by DNS colorimetry.

The specific steps are as follows: weigh 5.0 g of dried soil samples, put them into a test tube, and injected 15 mL of 8% sucrose solution, 5 mL of phosphoric acid buffer with pH 5.5 and 0.1 mL of toluene. 180R was shaken for 10 min, and then placed in an incubator for cultivation at 37℃ for 24h. Take it out at that time, centrifuge it at 6000 RPM for 10 min (or filter it), take 1 mL of supernatant into test tube, add 1.5 mL of 3, 5-dinitrosalicylic acid chromogener, boil it in water for 5 min, then cool it under tap water, and keep volume to 25 mL (the concentration is found to be too high in practice, 1 mL of 25 mL was absorbed and diluted 10 times for measurement) shaken well, and then colorimetric was performed on a spectrophotometer at 510 nm wavelength to convert the amount of glucose in 25 mL of the reaction solution. In order to eliminate the errors caused by the original sucrose and glucose in the soil, each soil sample should be made a non-matrix control (adding soil sample, without sucrose), and no soil control should be made, and the distilled water is relatively zero.

# Methods of soil physicochemical property detection

1. **Detection of soil pH**

2.00 g soil was accurately weighed, and 20 mL distilled water was accurately added according to the soil and water ratio (W/V), and then mixed. After that, the soil was placed on a 37 ℃ shaking table for 1 h. After that, the soil was removed and the soil pH was measured with a calibrated precision pH meter.

# Detection of soil total nitrogen

5 g Na2SO4 and 0.5 g CuSO4 were added to the bottom of the digestion tube. After heating, the sample was digested. After 4 hours, the sample was digested into gray green color. The digestion was continued for 2 h to make the ammonium salt reaction complete. After the digestion was completed, it was cooled and still to be distilled. Before distillation, it is necessary to check whether the distillation device is leaking, and add a small amount of deionized water into an empty boiling tube, use automatic liquid adding about 28 mL 35 % NaOH, add about 30 mL 1.5 % boric acid absorption solution into the absorption bottle, start the Kjeldahl apparatus through steam distillation, and use the distillate to clean the pipe. After the digestion and cooling of the liquid, the boiling tube was connected to the Kay nitrogen analyzer, and 28 mL 35% NaOH was added to make the solution strongly alkaline, and then insert 30 mL 1.5% boric-acid solution, insert the end of the condensing tube below 1 cm of the absorption solution, start the distillation with the Kay’s nitrogen apparatus until the distillate does not contain ammonia (the distillate does not have color reaction with the Natlerite reagent), stop the distillation, and wait for determination. 2~3 drops of bromocresol green - methyl red mixed indicator were added into the sample distillate, and the distillate was titrated with the calibrated hydrochloric acid standard solution to make it change from blue-green to red-purple. Record the volume of hydrochloric acid used (mL).

# Determination of available phosphorus in soil samples

Weigh 2.5 g (accurate to 0.001 g) of the air-dried sample passed through a 20-mesh sieve into a 150 mL triangular flask, add 50 mL of 0.5 mol/L NaHCO3 solution, shake it on an oscillating machine for 30 min, and immediately filter it with phosphorus-free

filter paper. Filtrate was taken from 100 mL triangular flask, 5 mL filtrate was absorbed, and 0.5 mol/L NaHCO3 solution was added to 10 mL in 150 mL triangular flask. Then 35 mL distilled water was added with burette, and 5 mL molybdenum antimony antireagent was added into pipette, and shaken well. After 30 min, colorimetry was performed at 880 nm. Take the absorption value of blank liquid as 0, read the absorption value of liquid to be tested.

# Determination of available potassium in soil samples

Preparation of potassium standard solution: 0.1907 g potassium chloride (analytical grade, dried at 110 °C for 2 h) was dissolved in 1 mol/L ammonium acetate solution, and the volume was set to 1 L making 100 µg/mL stock. The following standards were prepared using the stock: 0, 2.5, 5.0, 10.0, 15.0, 20.0, 40.0 µg/mL. The stock series solutions were prepared by pouring 0, 2.5, 5.0, 10.0, 15.0, 20.0 and 40.0 mL of the stock, respectively, into the 100 mL volumetric flask, followed by the addition of 1 mol/L ammonium acetate solution and filled with water to the final volume of 100 mL.

Working curve was prepared by measurement of each standard using flame photometer.

Sample analysis: weigh 0.5 g of air-dried sample (sieved by the 2 mm sieve), put it into the extraction flask, add 50 mL of 1 mol/L ammonium acetate solution, plug and shake for 30 min, filter with dry filter paper, and analyze the filtrate directly using flame photometer. The content of potassium was expressed as µg/kg basing on the working curve.

# Determination of soil organic matter in soil samples

Weigh 0.2 g (accurate to 0.0001 g) of the air dried sample that has passed the 1 mm sieve and place it in a 500 mL triangular bottle. Add 50.0 mL of 0.8 mol/L potassium dichromate solution accurately, then add 50.0 mL concentrated sulfuric acid, add a small curved neck funnel and place it in boiling water. Let the water boil for 30 minutes. Take it out and cool it to room temperature. Rinse the small funnel with water. The flask was removed, and the reactants were transferred to a 250 mL volumetric flask without loss, cooled to room temperature and constant volume. 50.0 mL solution was absorbed into the 250 mL flask, and water was added to about 100 mL, 3 drops of o- phenline indicator were added. When titration near the end point with 0.2 mol/L ferrous sulfate standard solution, and the solution changes from green to dark green, and a standard solution of ferrous sulfate is added drop by drop until brick red. At the same time, 0.2 g (accurate to 0.001 g) silica was substituted for the sample, followed the same analytical steps, and the same reagent was used for the blank test. If the amount of ferrous sulfate standard solution used for titrating sample is less than 1/3 of the amount of ferrous sulfate standard solution used for blank test, the sample weight should be reduced and re-measured.
